# Determinants of motorcycle helmet availability and cost in retail outlets: outcomes of a market survey in northern Ghana

**DOI:** 10.1186/s12889-023-15695-8

**Published:** 2023-04-26

**Authors:** Benjamin Noble Adjei, Emmanuel K. Nakua, Peter Donkor, John Amissah, Daniel Gyaase, Yeetey Enuameh, Charles Mock

**Affiliations:** 1 Department of Epidemiology and Biostatistics, Kwame Nkrumah University of Science and Technology, Kumasi, Ghana; 2 Department of Surgery, Kwame Nkrumah University of Science and Technology, Kumasi, Ghana; 3 Department of Environmental, Occupational Health and Safety, Kwame Nkrumah University of Science and Technology, Kumasi, Ghana; 4 Department of Surgery, University of Washington, Seattle, WA USA

**Keywords:** Motorcycle helmets, Helmet availability, Helmet cost, Retail outlets, Standard helmets, Northern Ghana

## Abstract

**Background:**

Morbidity and mortality from road traffic crashes are steadily increasing globally and they remain a major public health challenge. This burden is disproportionately borne by low-and middle-income countries, especially Sub-Saharan Africa where motorcycle helmet use is low and where there are challenges of affordability and availability of standard helmets. We sought to assess the availability and cost of helmets in retail outlets in northern Ghana.

**Methods:**

A market survey of 408 randomly sampled automobile-related retail outlets in Tamale, northern Ghana was conducted. Multivariable logistic regression was used to identify factors associated with helmet availability and gamma regression was used to identify factors associated with their cost.

**Results:**

Helmets were available in 233 (57.1%) of surveyed retail outlets. On multivariable logistic regression, street vendors were 48% less likely and motorcycle repair shops 86% less likely to sell helmets than automobile/motorcycle shops. Outlets outside the Central Business District were 46% less likely to sell helmets than outlets inside that district. Nigerian retailers were five times more likely to sell helmets than Ghanaian retailers. Median helmet cost was 8.50 USD. Helmet cost decreased by 16% at street vendors, 21% at motorcycle repair shops, and 25% at outlets run by the owner. The cost increased by older age of retailer (1% per year of age), education level of retailer (12% higher for secondary education, 56% higher for tertiary education, compared to basic education), and sex (14% higher for male retailer).

**Conclusion:**

Motorcycle helmets were available in some retail outlets in northern Ghana. Efforts to improve helmet availability should address outlets in which they are less commonly sold, including street vendors, motorcycle repair shops, outlets run by Ghanaians, and outlets outside the Central Business District.

**Supplementary Information:**

The online version contains supplementary material available at 10.1186/s12889-023-15695-8.

## Background

Morbidity and mortality from road traffic crashes are steadily increasing globally and they remain a major public health challenge [1, 2]. A significant proportion of these are due to motorcycle crashes. An estimated 3.4 million motorcycle crash deaths occurred between 2008 and 2020, of which 1.4 million could have been avoided if riders had used adequate protective gear [3]. This mortality burden is disproportionately borne by low-and middle-income countries (LMICs) [2], where motorcycles and bicycles are common means of transport [1]. A United Nations report indicated that the risk of death is 26 times higher for motorcyclists compared to drivers of cars in Europe [3]. In Ghana, the frequency of motorcycle crashes and fatalities has been on the rise in recent years and fatalities from motorcycle crashes exceeded that of pedestrians for the first time in 2020 [4,5].

Motorcycle helmets are one of the most effective safety interventions for motorcyclists, reducing the risk of severe head injuries, fatality, and associated costs by about half [6]. Helmet use improves the odds of survival from motorcycle crashes by 42% and helps avoid 69% of head injuries [3, 7]. Despite their protective effect, helmet use is low in many LMICs, due to factors, such as absent mandatory helmet laws, infrequent enforcement even when such laws exist [], low helmet availability and high cost [1]. The United Nations has described market forces that perpetuate high prices by maintaining a low demand and supply [3]. In addition, in many countries a high percentage of helmets are non-standard (e.g. half coverage, designed for other purposes, or damaged) and less protective [10,11,12]. However, they are frequently used in light of their lower costs as price is an important factor in helmet purchase considerations [13,14]. This explains why the use of non-standard helmets is common in most LMICs, including Ghana. A study conducted in nine LMICs revealed that standard helmets were between two and three times more expensive than non-standard helmets [13].

The affordability and availability of standard helmets remain a challenge in Ghana [13]. This poses a serious public health threat to motorcyclists as road traffic crashes continue to increase with increasing motorcycle use [1]. A limited number of studies have explored motorcycle helmets availability and cost in LMICs. In sub-Saharan Africa, most studies have focused on the prevalence of helmet use [8,9,15,16], not addressing helmet availability and cost. Furthermore, the few studies reporting on availability and cost were predominantly descriptive in nature [13,17], not examining factors that influence availability and cost. Therefore, this study aimed to assess the availability and cost of motorcycle helmets in retail outlets in northern Ghana, where motorcycles are the dominant mode of transportation and over 80% of families own motorcycles [18,19]. We also sought to assess factors associated with helmet availability and cost.

## Methods

### Study design and setting

An analytical cross-sectional survey of retail outlets of motorcycle/automobile related products in the Tamale Metropolitan area in the Northern Region of Ghana was conducted between March and April 2021. Bicycle and motorcycle ownership is over 80% in this area [19]. Most of the dwellers in and around the Central Business District use motorcycles as their means of transportation [18]. The principal streets and most linking roads in the city have side lanes for pedestrians and cyclists [18].

### Study population

The study units of interest were retail outlets selling automobile/motorcycle related products in the Tamale Metropolitan Area. Retail outlets that were situated more than 10 km away from the Central Business District of the Tamale Metropolis were excluded since there are limited economic activities around such areas.

### Sample size and sampling procedure

The desired sample size was estimated based on an anticipated 50% of retail outlets selling helmets, 5% margin of error, and 95% confidence level. The sample size was adjusted for a 10% non-response rate giving approximately 423 retail outlets to be surveyed.

The Tamale Metropolitan Area was purposively selected since most of the dwellers use motorcycles as their means of transportation. All retail outlets that sell automobile/motorcycle related products, including street market/vendors, along the highways and other linking roads and within a 10 km range around the Central Business District were listed. In all, 768 such retail outlets were listed. This includes outlets that sold only automobile related products and those that sold other merchandise also. Retail outlets that did not sell automobile related products were excluded. This constituted the sampling frame. A random number table was used to randomly select 423 retail outlets for the study.

### Data collection

The study employed trained research assistants to collect data from participants using a structured questionnaire. The respondents were interviewed face-to-face with an android tablet loaded with the questionnaire and enabled with Open Data Kit (ODK) [20]. The questionnaire contained information on helmet availability and costs as well as socio-demographic characteristics of respondents. Owners of the selected retail outlets were selected for the interviews. However, when the owner was not available at the time of data collection, the manager or the person in-charge of the outlet was interviewed. Available motorcycle helmets at the retail outlets were observed and assessed on recommended standards. Full-face helmets that were not damaged or cracked and that had certification markings were considered as standard [13]. Non-standard helmets on the other hand consisted of half-coverage or open-face helmets or any helmets that lacked certification markings or were damaged. A sample from each brand of helmet was inspected for the above characteristics and the data recorded. When an outlet did not have any helmet in stock at the time, interviewers obtained information on the brands that were usually sold. Data were not collected on helmets designed for other purposes, such as those for cycling or construction.

Interviewers also sought information on the average, least, and most expensive costs of the helmets. Data on costs were standardized in United States dollars using the exchange rate at the time of data collection. For retail outlets that did not have any helmet in stock at the time of the interview, costs of those usually sold were collected. A total of 12 research assistants were trained for 5-days on the study objectives, concepts, data collection procedures, completion of consent forms, and the knowledge and skills required to collect accurate and reliable data for the study.

### Study variables

The study employed two main outcome variables. The first was the availability of motorcycle helmets in the retail outlets, which was measured in a binary form: i.e., retail outlet did or did not sell motorcycle helmets. This included both standard and non-standard helmets as evidence from previous studies has shown that all types of helmets (standard or non-standard helmets) provide some level of protection for motorcyclists [10, 11]. The second outcome was the cost of standard helmets. Although all types of helmets provide some level of protection, standard helmets provide the highest level of protection [10]. Focusing on the cost of standard helmets conveys the importance of motorcycle safety, ensuring that motorcyclists have access to information on affordability of safe helmets, and motivating them to purchase helmets that offer the highest level of protection. The independent variables used for the analysis included the socio-demographic and other characteristics of the retailers and their businesses. Retailers’ characteristics such as age, sex, education level, religion, marital status, nationality, type, location, and ownership of retail outlet were purposively selected to explore the factors associated with helmet availability and cost. These factors could be used in efforts to promote increased availability or lower cost of helmets. It is purported that several factors related to helmets availability and cost (such as the ones addressed in the current study) need to be assessed to better understand country-specific problems to ensure successful helmet use programmes [].

### Statistical analysis

Data were summarized using frequencies and percentages for categorical variables. Mean, standard deviation, median and inter-quartile range were used to summarize continuous variables. Bivariate and multivariable logistic regression models were used to explore factors associated with helmet availability. Shapiro–Wilk test of normality showed that the cost of standard and non-standard helmets (measured as median cost for each helmet type at each retail outlet) were not normally distributed (p < 0.05). The gamma regression model with a log link and robust standard errors was used to model the factors influencing the cost of standard helmets. The gamma regression was used due to the highly skewed nature of helmet costs [21]. Exponents of the coefficients of the gamma regression [exp(β)] were reported. Variance Inflation Factor (VIF) was computed to check for multicollinearity. Variables significant at the p < 0.1 level in bivariate analysis were included in the multivariable regressions. Confidence intervals were computed at a 95% confidence level and a p-value ≤ 0.05 was considered as statistically significant for the final models. Data were analyzed using Stata 16.0.

### Ethical consideration

Ethical clearance was obtained from Committee on Human Research Publication and Ethics (CHRPE/AP/128/21), Kwame Nkrumah University of Science and Technology. Additional administrative approval was obtained from the Tamale Metropolitan Assembly. Consent to participate in the study was obtained from the respondents who were the managers or attendants on duty of the retail outlets. Legally Authorized Representatives of illiterate participants provided informed consent for the study. Again, informed consent was obtained from parents and/or legal guardians of participants who were less than 18 years for study participation. The consent process included verbally explaining the purpose of the study, the risks, benefits and assuring the participants of confidentiality and anonymity. A consent form was given to those who agreed to participate to either sign or thumbprint. Data collected from the respondents were de-identified and stored on a cloud-based server with passwords known to the principal investigator.

## Results

In all, 423 retail outlets that had automobile/ motorcycle-related products were contacted, out of which 408 consented, yielding a 96.5% response rate. As per the findings in Table 1, the mean age of the respondents was 31.2 ± 9.1 years. Majority (81.4%) of the respondents were males. Majority (83.3%) had ever been to school. Of those who had ever gone to school, most (66.2%) had Senior High School education. A little more than half (54.4%) of the respondents were married, 68.1% were Muslims, and 64.5% were Dagombas (the main ethnic group in Tamale) while 21.3% were of the Igbo/Yoruba ethnicity of Nigeria.

**Table 1:** Socio-demographic characteristics of respondents

Variable	Frequency (*n* = 408)	Percent (%)
**Age (years)**		
**Mean ± SD**	31.2 ± 9.1	
< 20	32	7.8
20 – 29	163	40.0
30 – 39	149	36.5
40 – 49	44	10.8
50 +	20	4.9
**Sex of respondent**		
Female	76	18.6
Male	332	81.4
**Ever been to school**		
No	68	16.7
Yes	340	83.3
**Level of education (*n* = 340)**		
Basic school	70	20.6
Senior high school	225	66.2
Tertiary	45	13.2
**Marital status**		
Single (never married)	183	44.9
Married (registered partner)	222	54.4
Divorced/separated/widowed	3	0.7
**Religion**		
Muslim	278	68.1
Christian	130	31.9
**Nationality**		
Ghanaian	321	78.7
Nigerian	87	21.3
**Ethnicity**		
Dagomba	263	64.5
Gonja	15	3.7
Akan	23	5.6
Igbo/Yoruba	87	21.3
Others^a^	20	4.9

^a^Grusi, Mamprusi, Moshi, Sissala

### Availability of motorcycle helmets in the retail outlets

Motorcycle helmets were available in 233 (57.1%) of the surveyed retail outlets. Of the outlets that sold helmets, most (182, 78.1%) sold only standard helmets. But some (31, 13.3%) sold both standard and non-standard helmets and a few (20, 8.6%) sold only non-standard helmets. The median number of helmet brands sold was 2 (min = 1, max = 12). In all, 572 different helmets were observed/inspected. These were of 59 different brands, all of which were imported into Ghana. Of the 572 helmets inspected, 501 (87.6%) were standard while 71 (12.4%) were non-standard. Most outlets (83.7%) had helmets in stock during the time of the survey, however, 71.2% had ever run out of stock of motorcycle helmets. More than half (64.4%) of the outlets had their wholesale supply of helmets from Tamale (Table 2).

**Table 2:** Availability of motorcycle helmets in the retail outlets

Variable	Frequency (*n* = 408)	Percent (%)
**Type of retail outlet**		
Automobile/motorcycle shop	187	45.8
Informal road/street market	87	21.3
Supermarket	70	17.2
Motorcycle repair shop	64	15.7
**Was respondent the owner of the outlet**		
No	191	46.8
Yes	217	53.2
**Sells motorcycle helmets**		
No	175	42.9
Yes	233	57.1
**Number of different brands of helmets sold (*n* = 233)**		
One	75	32.2
2–3	120	51.5
4 +	38	16.3
**The average number of helmets sold in a day (*n* = 233)**		
None	55	23.6
One	67	28.7
2–3	71	30.5
4 +	40	17.2
**Helmet currently in stock (*n* = 233)**		
No	38	16.3
Yes	195	83.7
**Ever run out of stock (*n* = 233)**		
No	67	28.8
Yes	166	71.2
**Frequency of running out of stock (*n* = 166)**		
Most of the time	38	22.9
Once a while	128	77.1
**Place of purchasing wholesale helmets**		
Tamale (*n* = 233)	150	64.4
Kumasi (*n* = 233)	82	35.2
Accra (*n* = 233)	94	40.3
Outside Ghana (*n* = 233)	71	30.5
**All motorcycle helmets sold in the retail outlet are of the recommended standard (*n* = 233)**		
No	51	21.9
Yes	182	78.1
**Number of brands of standard helmets in a retail outlet (*n* = 233)**		
None	20	8.6
One	67	28.7
2–3	109	46.8
4 +	37	15.9
**Number of brands of non-standard helmets in a retail outlet (*n* = 51)**		
One	37	72.5
2–3	13	25.5
4 +	1	2.0

### Factors associated with motorcycle helmet availability in the retail outlets

Factors associated with helmet availability in the retail outlets were explored using bivariate and multivariable logistic regression models. The bivariate model revealed that the type and location of the retail outlet and the sex, educational level, religion, and nationality of the respondents significantly influenced the availability of motorcycle helmets. In the multivariable model, informal road vendors/street markets were 48.0% less likely to sell motorcycle helmets compared to automobile/motorcycle shops [AOR = 0.52, 95%CI = 0.28–0.98] and motorcycle repair shops were 86.0% less likely to sell helmets compared to automobile/motorcycle shops [AOR = 0.14, 95% CI = 0.06–0.34]. Retail outlets owned by Nigerians were five times more likely to sell motorcycle helmets compared to outlets owned by Ghanaians [AOR = 5.08, 95%CI = 2.13–12.12]. Retail outlets outside the Central Business District were 46.0% less likely to sell helmets compared to outlets within that district [AOR = 0.54, 95%CI = 0.33–0.88] (Table 3).

**Table 3:** Factors associated with helmet availability in the retail outlets

Variable	COR	95%CI	*p*-value	AOR	95%CI	*p*-value
**Type of retail outlet**						
Automobile/motorcycle shop						
Informal road/street vendor	0.58	0.34 – 0.98	0.041	**0.52**	**0.28 0–0.98**	**0.044**
Supermarket	0.68	0.38 – 1.20	0.184	0.52	0.26 – 1.07	0.077
Motorcycle repair shop	0.07	0.03 – 0.16	0.001	**0.14**	**0.06 – 0.34**	**0.001**
**Location of retail outlet**						
Within CBD						
Outside CBD	0.51	0.34 – 0.76	0.001	**0.54**	**0.33 – 0.88**	**0.014**
**Age (years) of respondent**						
< 20						
20 – 29	1.87	0.88 – 3.98	0.104			
30 – 39	1.99	0.93 – 4.27	0.075			
40 – 49	1.17	0.47 – 2.88	0.737			
50 +	1.27	0.42 – 3.82	0.63			
**Sex of respondent**						
Female						
Male	0.56	0.33 – 0.94	0.030	0.69	0.38 – 1.28	0.277
**Ever been to school**						
No						
Yes	1.14	0.68 – 1.92	0.616			
**Level of education**						
Basic school						
Senior high school	2.65	1.53 – 4.58	0.001	1.55	0.81 – 2.97	0.185
Tertiary	2.58	1.20 – 5.52	0.015	1.79	0.75 – 4.25	0.188
**Marital status**						
Single (never married)						
Married (registered partner)	0.99	0.67 – 1.47	0.974			
Divorced/separated/widowed	0.45	0.06 – 3.45	0.440			
Religion						
Muslim						
Christian	3.39	2.13 – 5.40	0.001	1.43	0.64 – 3.21	0.383
**Nationality**						
Ghanaians						
Nigerians	6.95	3.60 – 13.40	0.001	**5.08**	**2.13 –12.12**	**0.001**

*COR* Crude Odds Ratio, *AOR* Adjusted Odds Ratio, *CI* Confidence Interval, *CBD* Central Business District, Hosmer–Lemeshow goodness-of-fit test (χ2 = 68.62, *p* = 0.623)

### Cost of motorcycle helmets in the retail outlets

The median cost of standard helmets was 8.50 (min = 6.80, max = 81.60) USD and that of non-standard helmets was 6.80 (min = 5.10, max = 25.50) USD. Most (72.1%) of the retail outlets sold standard helmets between 8.50 to 17.00 USD while majority (90.2%) sold non-standard helmets below 8.50 USD. The median cost of the cheapest helmet (standard or non-standard) in each outlet was 7.65 (min = 5.10, max = 51.00) USD and that of the most expensive helmet (standard or non-standard) was 11.90 (min = 5.95, max = 81.60) USD. Most (66.9%) of the retail outlets sold the most expensive helmet (standard or non-standard) between 8.50 and 17.00 USD (Table 4). The cost of helmets must be put into the context of the local economy. The average daily wage in Ghana is 5 USD [22]. Hence, the median cost of a standard helmet represented 1.5 days wages.

**Table 4:** Cost of motorcycle helmets in the retail outlets

Variable	Frequency (*n* = 233)	Percent (%)
**Median cost of standard helmet in each outlet (USD) [*n* = 213]**		
< 8.50	49	23.0
8.50 – 17.00	149	70.0
> 17.00	15	7.0
**Median cost of non-standard helmet in each outlet (USD) [*n* = 51]**		
< 8.50	46	90.2
8.50 – 17.00	4	7.8
> 17.00	1	2.0
**Cost of cheapest helmet (USD)**^a^		
< 8.50	120	51.5
8.50 – 17.00	107	45.9
> 17.00	6	2.6
**Cost of most expensive helmet (USD)**^a^		
< 8.50	16	6.9
8.50 – 17.00	156	66.9
> 17.00	61	26.2

*USD* United States Dollar

^a^Standard or non-standard

### Factors associated with the average cost of standard helmets in the retail outlets

Table 5 presents the gamma regression analysis showing the factors associated with the average cost of standard helmets in the retail outlets. The type of retail outlet, and the sex, level of education, and ownership status of the retailer were significantly associated with helmet cost on bivariate analysis. In the adjusted model, the average cost of a standard helmet was estimated to be less by 16.0% when purchased from informal road/street vendors compared to automobile/motorcycle shops [exp(β) = 0.84, 95%CI = 0.76–0.93] and less by 21.0% from motorcycle repair shops compared to automobile/motorcycle shops [exp(β) = 0.79, 95%CI = 0.67–0.93]. Also, each one year increase in the age of retailers was estimated to increase the cost of a standard helmet by 1.0% [exp(β) = 1.01, 95%CI = 1.00–1.03] whilst male retailers were estimated to increase the cost of a standard helmet by 14.0% [exp(β) = 1.14, 95%CI = 1.01–1.29]. The cost of standard helmets was estimated to increase by 12.0% and 56.0% for retailers with senior high and tertiary education compared to those with basic education [exp(β) = 1.12, 95%CI = 1.01–1.23; exp(β) = 1.56, 95%CI = 1.12–2.19] respectively. The cost of a standard helmet was estimated to decrease by 25.0% at outlets run by the owner compared to outlets run by someone other than the owner [exp(β) = 0.75, 95%CI = 0.63–0.88]. The actual cost vs. the cost predicted from the gamma regression is shown in Fig. 1 (supplementary file).

**Table 5:** Factors associated with the average cost of standard helmets in retail outlets

	**Unadjusted**			**Adjusted**		
**Variable**	**exp (β)**	**95%CI**	***p*-value**	**exp (β)**	**95%CI**	***p*-value**
**Type of retail outlet**						
Automobile/Motorcycle shop						
Informal road/street vendor	0.73	0.65 – 0.83	0.001	**0.84**	**0.76 – 0.93**	**0.001**
Supermarket	0.94	0.67 – 1.30	0.707	0.91	0.71 – 1.15	0.426
Motorcycle repair shop	0.73	0.64 – 0.82	0.001	**0.79**	**0.67 – 0.93**	**0.005**
**Location of retail outlet**						
Within CBD						
Outside CBD	1.15	0.96 – 1.39	0.135	1.10	0.96 – 1.27	0.158
**Age (years) of respondent**^a^	1.01	0.99 – 1.02	0.105	**1.01**	**1.00 – 1.03**	**0.026**
**Sex of respondent**						
Female						
Male	1.17	1.00 – 1.38	0.043	**1.14**	**1.01 – 1.29**	**0.030**
**Level of education**						
Basic school						
Senior high school	1.16	1.04 – 1.28	0.005	**1.12**	**1.01 – 1.23**	**0.027**
Tertiary	1.92	1.32 – 2.79	0.001	**1.56**	**1.12 – 2.19**	**0.009**
**Religion**						
Muslim						
Christian	0.76	0.65 – 0.89	0.001	0.91	0.81 – 1.03	0.143
**Nationality**						
Ghanaian						
Non-Ghanaian	0.77	0.67 – 0.89	0.001	0.90	0.80 – 1.02	0.108
**Was respondent the owner of the retail outlet**						
No						
Yes	0.83	0.69 – 1.01	0.058	**0.75**	**0.63 – 0.88**	**0.001**
**All helmets sold are standard helmet**						
No						
Yes	1.08	0.91 – 1.29	0.354			

exp (β)- Exponent of the coefficient (β) of the gamma regression model, *CBD*  Central Business District, Deviance goodness-of-fit test (deviance = 29.10, *p* = 1.00), ^a^Age was considered as both categorical and linear specification but the model with age as linear specification performed better than the one with the age as categorical (AIC = 2391.14 vs. 2396.72)

## Discussion

This study highlights the availability and cost of motorcycle helmets in retail outlets in northern Ghana and the factors associated with their availability and cost. Helmets were available in about 6 in 10 retail outlets. Type of retail outlet, nationality of the retailer, and location of the outlet were significantly associated with helmet availability. Standard helmets were sold above the average daily wage in Ghana. The factors that influenced the cost of standard helmets were the type and location of the retail outlet, and the age, sex, and level of education of the retailers.

This is one of the few studies addressing the availability of motorcycle helmets in retail outlets in an LMIC such as Ghana. The findings suggest helmets were available in about half of the surveyed retail outlets, which agrees with the few previous studies on this topic in LMICs [14]. It has been argued that low availability of helmets is associated with high cost of helmets, which in turn will lead to low utilization. Inversely, low utilization will also result in low availability [3]. It is therefore likely that the limited availability of helmets in the current study is in part due to low utilization of helmets amongst motorcyclists as reported by previous studies in Ghana [8, 9, 23].

Moreover, the current study revealed that about 2 in 10 helmets found in the retail outlets were not of the recommended standard. This is similar to a study conducted in Nigeria which suggested about 4 in 10 of the helmets used by motorcyclists were sub-standard [16]. Similarly, Ackaah et al. found that non-standard helmets were widely used in LMICs [13]. The availability of appropriate helmets in LMICs is an issue of great concern. Inferior sub-standard helmets might not provide adequate protection. The United Nations Motorcycle Helmet Study concluded that progress made towards helmet availability should not compromise on the quality of helmets [3]. The utilization of sub-standard helmets largely depends on their cost and availability in the markets. If these inferior sub-standard helmets, with their associated low cost, are continuously available in the market, the use of non-standard helmets by motorcyclists would increase, which could pose a challenge to the achievement of the Sustainable Development Goal target of halving the global number of injuries and deaths from road traffic accidents by 2030 [10, 24]. While all types of helmets offer some degree of protection, standard helmets offer the most optimal level of protection [10]. Therefore, helmets that meet recommended standards should be sold in retail outlets at affordable prices []. Although, majority of the retail outlets had helmets in stock during the time of the survey, more than two-thirds of these outlets had ever run out of stock of motorcycle helmets. The reason for the stock outs is not apparent, however, it is evident that all motorcycle helmets are imported. Therefore, efforts to improve helmet availability should promote local manufacturing given the taxes on importation.

In this study, the majority of outlets sold standard helmets between 8.50 USD to 17.00 USD whilst most (90.2%) sold non-standard helmets below the minimum price of a standard helmet. Ackaah et al. found that the cost of helmets for most retail outlets in Ghana was 20 USD and above in 2012 [13], which is higher than the cost found in the current study. The difference from the current study might reflect fluctuations in exchange rates or an actual decrease in the price. In Mexico, it was found that the average cost of a standard helmet was 124 USD which is far higher than the current study [17]. The median cost of a standard helmet of 8.50 USD in the current study represents the equivalent of 1.5 days average wage, which is substantial. In a study of the cost of safety devices in 18 countries, Hendrie et al. showed that bicycle helmets ranged in cost from 1 hour wage in Austria to 18 hours (over 2 days) wage in Albania. Helmets in most lower income countries (e.g. Vietnam, Venezuela) cost the equivalent of 12–18 hours wages, whereas helmets in most higher income countries (e.g. US, Western Europe) cost 1–10 hours wages [14]. Hence, the costs of helmets in the current study are commensurate with those in other LMICs. Costs are lower than 10 years ago in Ghana, but still represent a hardship for many people, especially those with lower incomes. Prices of helmets are largely determined by retailers as there is no legislation in Ghana that regulates prices of such commodities. This therefore gives retailers the opportunity to sell helmets at uncontrolled prices without any reasonable upper or lower bounds for the cost of these helmets. Efforts to further decrease the costs of helmets are warranted.

This is one of the few studies that employed an in-depth analysis using rigorous statistical methods to determine factors influencing the cost of standard helmets. The cost of standard helmet was less at informal road/street markets compared to automobile/motorcycle shops. Some factors contributing to increasing costs were older age and higher educational level of the retailers. The reason for this is not immediately apparent but may indicate that outlets run by older more educated people tend to be the higher end, more expensive stores. Helmet availability was higher amongst Nigerian retailers as compared to Ghanaian retailers. The Nigerians in Northern Ghana are noted for selling automobile-related products, but there is no a priori reason why automobile-related outlets run by Nigerians should be more likely to sell helmets than those managed by Ghanaians. Helmet promotion should focus on increasing the availability of helmets in outlets run by Ghanaians. These and other factors explored in this study could be used in efforts to promote increased availability or lower cost of helmets.

This study has emphasized the cost of standard helmets. By so doing, we hope to ensure that all motorcyclists have access to information on the affordability of safe helmets. This can lead to greater awareness and understanding of the potential risks of wearing substandard helmets, encouraging riders to prioritize safety over cost when purchasing a helmet. Moreover, promoting the use of standard helmets can motivate riders to invest in helmets that provide the best possible protection, thereby reducing the risk of head injuries in the event of a crash. To tackle the issue of high costs for standard helmets, it is important to consider the reduction of tariffs for imported helmets. Additionally, there is a need to investigate and address the reasons why some stores sell standard helmets at a higher price than others.

This study has several limitations. First, the study did not evaluate availability and cost of other types of helmets, such as helmets designed for other purposes (e.g. construction helmets). Some motorcyclists in Ghana and other LMICs use such helmets. Second, information on frequency of past stock outs, was based on self-reporting by the respondents and there was no way to verify the answers. Despite these limitations, this study has several strengths. It is one of the few studies addressing availability and cost of motorcycle helmets in retail outlets in an LMIC setting. Also, the use of statistical models to determine factors that influenced helmet availability and cost is novel and brings to light several factors which have not been previously explored.

## Conclusion

Motorcycle helmets were available in about 6 in 10 retail outlets in northern Ghana; however, non-standard helmets, which have the potential of undermining the benefits of helmets, were also available in some outlets. The availability of motorcycle helmets in retail outlets was significantly influenced by the type of retail outlet, nationality of the retailer, and location of the outlet. Standard helmets were sold above the average daily wage in Ghana and cost was influenced by type of retail outlet, ownership of retail outlet, age, sex, and education level of the retailer. The high cost of motorcycle helmets can have significant consequences for public health and safety, highlighting the need for efforts to increase access to affordable, high-quality helmets. Efforts to address the cost of standard helmets should consider relaxation on tariffs for helmets, as all of the helmets in the current study were imported. Efforts to improve helmet availability in the study area could also reasonably address increasing their availability in outlets in which they are less commonly sold (e.g. informal road/street vendors, motorcycle repair shops), outlets run by Ghanaians, and retail outlets located outside the Central Business District.

## Supplementary Information

**Additional file 1: Figure 1.** Actual versus predicted costs of standard helmets.

## Data Availability

The data used for this study are available upon reasonable request from the corresponding author.

## Abbreviations

AOR: Adjusted Odds Ratio
CBD: Central Business District
CHRPE: Committee on Human Research Publication and Ethics
CI: Confidence interval
COR: Crude Odds Ratio
LMICs: Low-and middle-income countries
ODK: Open Data Kit
SD: Standard Deviation
USD: United States Dollar
VIF: Variance Inflation Factor
