# Supplementary material for: Determinants of motorcycle helmet availability and cost in retail outlets: outcomes of a market survey in northern Ghana
Source: BMC Public Health. 2023 Apr 26;23:771. doi: 10.1186/s12889-023-15695-8 (PMC10131362; doi:10.1186/s12889-023-15695-8)
Supplement: Supplementary file 1 — Additional file 1: Figure 1. Actual versus predicted costs of standard helmets. [file 12889_2023_15695_MOESM1_ESM.docx]

Figure 1: Actual versus predicted costs of standard helmets
